# Is the Presence of Sinus Tract in Periprosthetic Joint Infection Still One of the Main Deciding Factors for Septic Revision?

**DOI:** 10.7759/cureus.75379

**Published:** 2024-12-09

**Authors:** Emanuel-Cristian Sandu, Adrian Cursaru, Bogdan Serban, Sergiu Iordache, Mihai Aurel Costache, Catalin Cirstoiu

**Affiliations:** 1 Orthopaedics and Traumatology, "Carol Davila" Faculty of Medicine, Bucharest, ROU; 2 Orthopaedics and Traumatology, University Emergency Hospital, Bucharest, ROU

**Keywords:** differential diagnosis pji, periprosthetic joint infection (pji), pji diagnosis, revision arthroplasty, septic loosening, sinus tract

## Abstract

Introduction

Two of the most common complications of joint arthroplasty surgery are aseptic and septic loosening. While aseptic loosening has a well-established treatment protocol, and diagnosis is quite straightforward, bacterial colonization of the implants is associated with a more difficult diagnosis and treatment, more surgeries, and higher morbidity for the patient. Accurate diagnosis is essential in choosing the right treatment plan. The aim of the current study was to assess the current diagnostic methods for periprosthetic joint infection and the influence of clinical signs like sinus tract on the treatment algorithm and outcomes of the patients. We wanted to highlight that sinus tract is still one of the major criteria in periprosthetic joint infection diagnosis and its presence increases the probability of choosing the right therapeutic option.

Materials and methods

During the three-year period of the study, we included 48 cases of patients who presented in our hospital with pain around their hip or knee prostheses. Inclusion criteria were patients diagnosed with septic or aseptic loosening of the prosthesis that required surgical revision of the implant in one stage or two stages. We excluded patients who did not require surgery yet or had major contraindications for revision surgery, patients who refused surgery, acute periprosthetic joint infections (less than 1 month since implantation), or extrinsic mechanical complications of the prosthesis like periprosthetic fractures, implant dislocations.

Results

Out of 48 patients, 25 underwent one-stage revision and 23 underwent two-stage revision surgery (septic revision). In the subgroup of two-stage revision, 18 patients (78.2%) presented a sinus tract communicating with the prosthesis, this clinical sign being a major characteristic of the subgroup. We managed to successfully identify 21 out of 23 cases (91.3%) of periprosthetic joint infections prior to or during the surgery. In the two cases in which we misdiagnosed the infection, the sinus tract or a positive bacterial culture was absent prior to surgery, in addition to other clinical or paraclinical findings indicating only a small probability of periprosthetic joint infection, influenced the attending medical doctor's therapeutic decision. In these particular cases of culture-negative periprosthetic joint infections, the outcome was poor, with patients needing additional surgeries in order to eradicate the infection.

Discussion

When present, a clear sign of periprosthetic joint infection, such as a sinus tract, facilitates the diagnostic protocol and allows the medical staff to initiate the appropriate treatment earlier. In the absence of such obvious signs, differential diagnosis remains difficult, and we should consider the future development of faster, cheaper, and more accurate tests for periprosthetic joint infection diagnosis, especially for chronic low-grade infections that could be easily misdiagnosed.

## Introduction

Periprosthetic joint infection (PJI) is considered one of the most serious reasons for implant degradation, on the one hand, because of the difficulty of establishing a fast and accurate diagnostic, and on the other hand, due to the laborious treatment and reserved prognostic [1]. For many years, there was no definitive diagnostic protocol accepted worldwide. Every scientific researcher or society used their own “gold standard” approaching this pathology, including clinical findings, laboratory data, radiological imaging, or data provided by microbiological or histopathological tests as the diagnostic criteria [2]. Today, the most used diagnostic protocols are represented by the ones proposed by the European Bone and Joint Infection Society (EBJIS), the Infectious Diseases Society of America (IDSA), and the Musculoskeletal Infection Society (MSIS) (2018 updated version).

Despite establishing a rigorous diagnostic protocol, differential diagnosis still remains an important challenge, with misdiagnosed periprosthetic joint infections having an impressive impact not only on the health and mobility of the patients but also on the healthcare system, considering the high financial costs and resources consumed managing such cases [3].

The presence of a communicating sinus tract with the articular prosthesis was always considered a major criterion for PJI diagnosis. All the aforementioned societies are using this clinical finding in their diagnostic protocols [4]. Although when present, the sinus tract can remove any doubt about the pathology we are facing, making it easier for the surgeon to choose the correct treatment plan, some studies have shown that it can be an inconsistent clinical sign of PJI. In a multi-center study published by Preobrazhensky et al. in 2020, we can observe that a sinus tract was present in only 20% of the patients who had a septic surgical revision of the arthroplasty [5].

The aim of the current study is to show the importance of an accurate and fast preoperative or intraoperative diagnostic of PJI and its correlation with patient evolution and success of arthroplasty revision surgery. In the case of septic revision, sometimes the definitive diagnosis is obtained following surgery only after pathogen bacteria is identified from cultures inoculated with samples harvested during surgery or a positive histopathological exam is conducted [6] confirming any preoperative suspicion. However, that leaves the medical team with limited therapeutic options, such as adjusting the dose, type, or period of antibiotic therapy according to antibiotic sensitivity testing and the virulence of the bacteria. We described more extensively the cases where the diagnosis was uncertain and observed the different outcomes of those patients.

## Materials and methods

Study design and population

We investigated in a retrospective manner data from 48 patients who underwent revision surgery of hip or knee arthroplasty in our hospital (University Emergency Hospital, Bucharest, Romania) for septic or aseptic loosening in the time frame of January 2018 - December 2020. We included patients with one-stage revision surgeries and two-stage revision surgeries that had a follow-up of at least one year. We excluded patients who did not require surgery yet or had major contraindications for revision surgery like sepsis, ASA type IV (American Society of Anesthesiologists classification), severe cardiovascular disease, kidney or liver failure, or patients who refused surgery, had acute periprosthetic joint infections (less than 1 month since implantation) treated with the Debridement, Antibiotics and Implant Retention (DAIR) procedure, or extrinsic mechanical complications of the prosthesis like periprosthetic fractures or implant dislocations.

**Figure 1:**
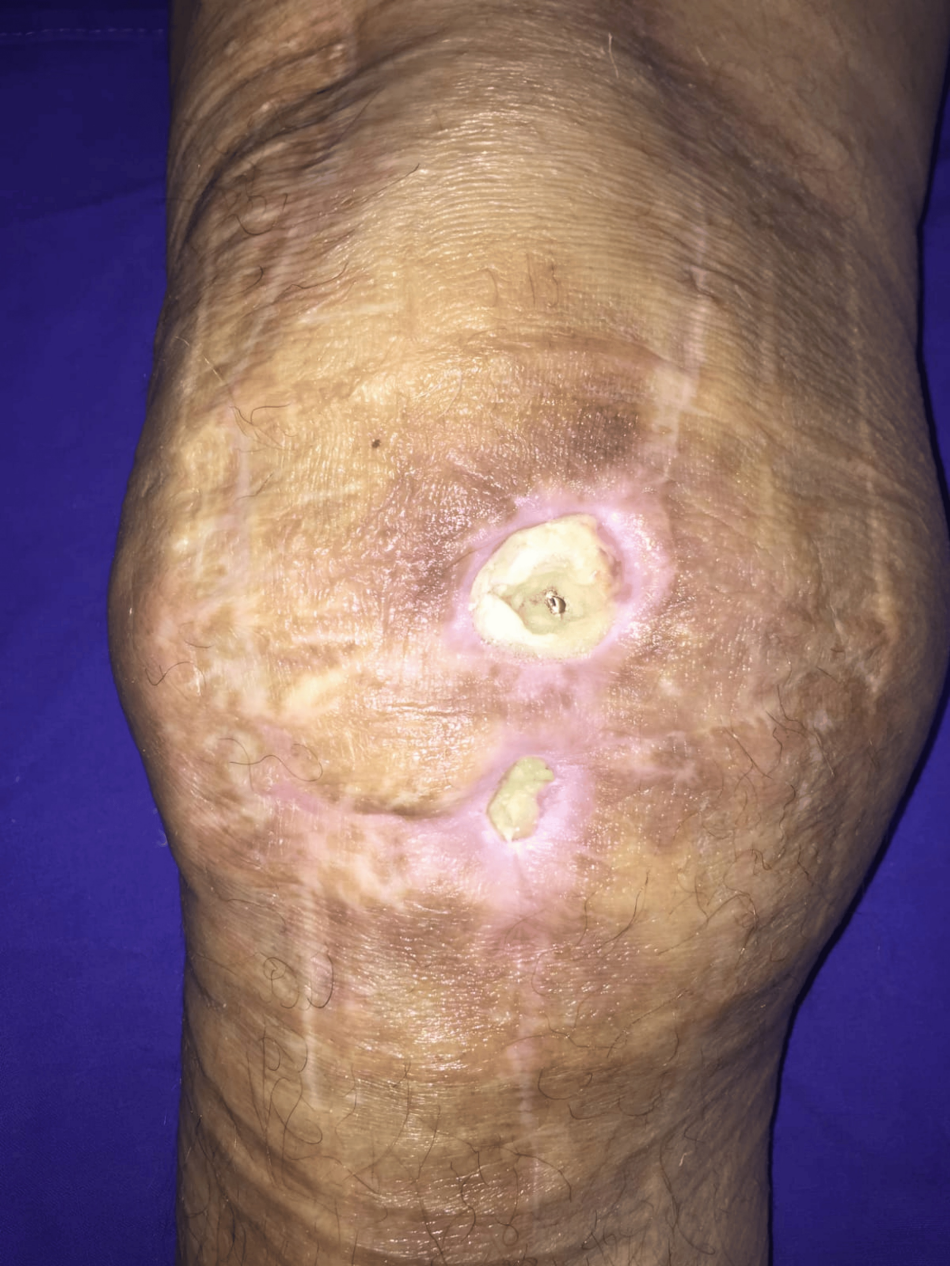
Anterior sinus tract overlapping the surgical incision and exposing the femoral component of the knee prosthesis

First, we divided the studied population into two subgroups: patients who underwent one-stage revision (n=25) and patients who underwent septic revision surgery in two stages (n=23). Being a multifactorial diagnostic, PJI was established with a major finding, such as sinus tract (Figure 1); purulence around the prosthesis; positive bacterial growth in synovial fluid, sonication fluid, or at least in two tissue samples; inflammation in periprosthetic tissue visualized in a histopathological exam; or meeting more cumulative minor criteria such as a value over 10 mg/L for C reactive protein (CRP), or over 30 mm/H for erythrocyte sedimentation rate (ESR); a history of joint or systemic infection, radiological osteolysis, or periosteal reaction in the first two or three years after primary arthroplasty; elevated synovial white blood cell; and more. Great emphasis was placed on data that could be obtained preoperative or intraoperative and could decide the course of treatment.

Treatment options

The major goals of PJI treatment are to restore the mobility of the joint, lower the morbidity of the patients, and certainly eliminate the infectious process. The treatment plan should be individualized and conducted by a multi-disciplinary team in order to achieve the best results [7]. An appropriate surgery and adjuvant antibiotic therapy are required for a successful treatment.

When we talk about arthroplasty revision surgery, there are three major concepts when approaching this pathology depending on the time elapsed from the primary prosthesis implantation, the extent of the infection, and the quality of the bone [8].

Debridement, Antibiotics and Implant Retention (DAIR)

Even though early studies regarding this strategy when approaching PJI showed high failure rates of the treatment [9], the success rates can be further improved when some conditions are met, such as a stable implant, a pathogen bacteria with good antibiotic response, no sinus tract or compromised soft tissue present, and a symptoms duration of less than 3 weeks.

One-Stage Implant Replacement

As the name says, one-stage implant replacement involves only one surgery for the revision of the prosthesis, involving the removal of the old one and reimplantation of a new prosthesis in one step. One-stage exchange is suitable for patients who have good bone conditions and soft tissue without sinus tract, as well as known bacteria with no difficult-to-treat infections caused by pathogens resistant to biofilm-active antimicrobials [10]. Even though this type of approach can be successfully used in both septic (when some criteria are met) and aseptic loosening, in our clinic, we unanimously accepted that this method is more suitable for aseptic loosening, mainly because most of our septic patients do not seek medical advice until the infectious disease evolution exceeds the limitations of this type of procedure.

Two-Stage Implant Replacement

**Figure 2:**
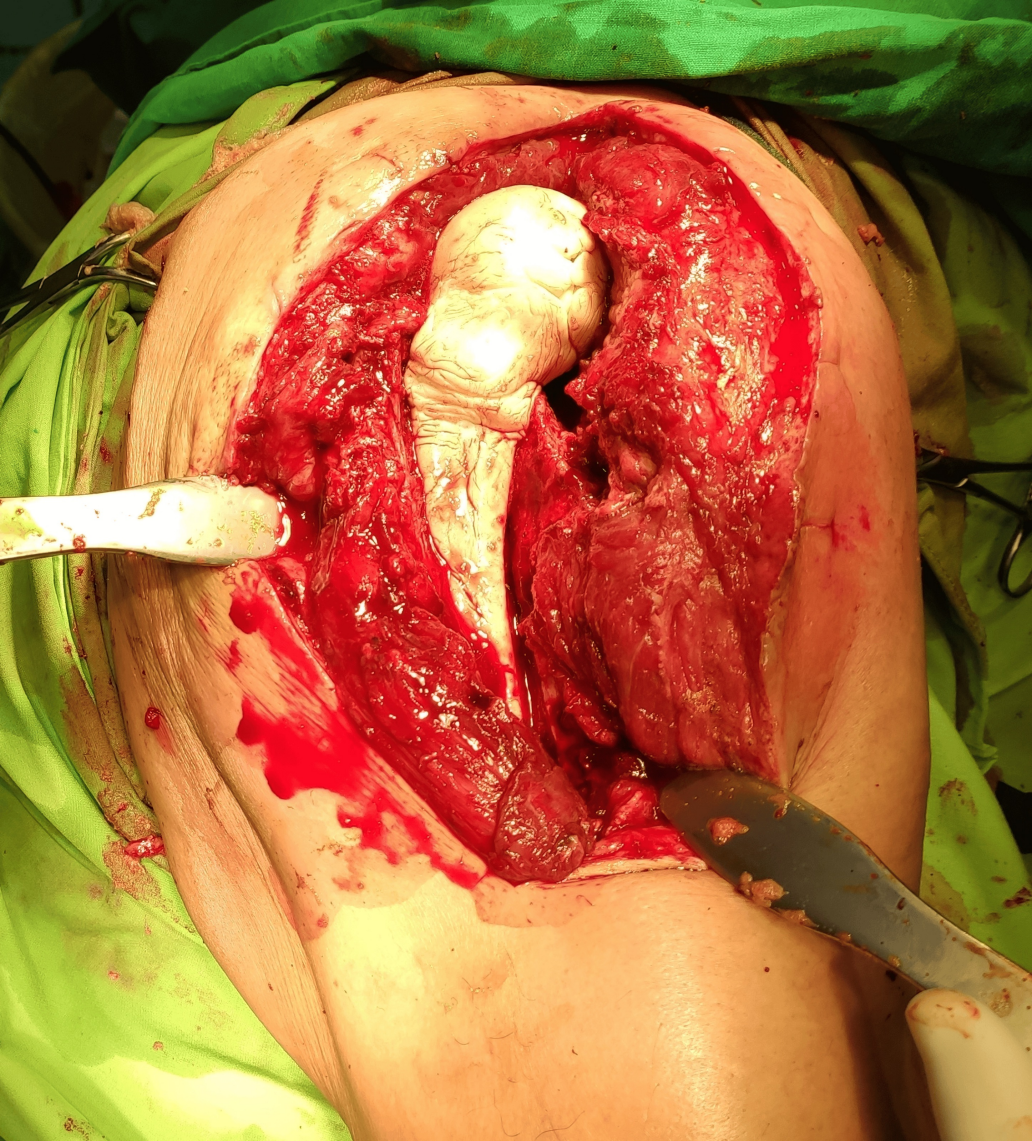
Gentamicin impregnated polymethylmethacrylate joint spacer implantation during hip arthroplasty revision surgery

This approach involves the removal of the prosthesis and implantation of a joint spacer in the first surgery and subsequently a delayed reimplantation of revision prosthesis in a second surgery. The period of time between the two surgeries can vary, from 4 to 8 weeks depending on the antibiotic susceptibility of the bacteria, resistant strains requiring prolonged treatment, and the condition of soft tissue [11]. While waiting for sanitation of the septic process, we implant a polymethylmethacrylate joint spacer impregnated with antibiotics (Figure 2) that will slowly be released locally in order to increase the local concentration of antibiotics and to preserve the length and mobility of the affected limb. Additionally, in cases where the pathogen bacteria are known to have a lower response to the treatment or the soft tissue is severely compromised, we implant absorbable calcium sulfate beads that carry and release antibiotics locally [12]. The two-stage exchange is considered the golden standard in treating PJI, mostly in patients with locally extensive infections [13], with reinfection rates after this type of procedure being slightly lower than one-stage revision [14].

Statistical analysis

Data were collected using Excel 6.2.14 (v16.0) (Microsoft, Redmond, WA, USA) and statistical analysis was performed using SPSS Statistics 21 (IBM Corp., Armonk, NY, USA). Chi-square and Mann-Whitney U tests were performed for hypothesis testing regarding the distribution of the variables in two groups. A p-value lower than 0.05 was considered to be statistically significant. The confidence interval was set at 95%.

## Results

Clinical and paraclinical findings

The most relevant data of our study is presented in Table 1. It is easy to observe that the defining characteristic of patients who underwent two-stage revision surgery is the presence of a sinus tract. Eighteen patients in this group (78.2%) had an active sinus tract communicating with the prosthesis of the hip or knee at the initial evaluation. The presence of a sinus tract was always correlated with two-stage revision surgery (p<0.001), and none of the patients from the one-stage revision group revealed this sign at the initial evaluation. Even though 23 cases of PJI were identified in the end, we can notice that only 21 cases (91.3%) were diagnosed prior to or during surgery and treated accordingly using the two-stage revision method because two cases were misdiagnosed and treated as aseptic loosening cases. 

**Table 1:** Population data distribution by revision surgery type CRP-C reactive protein, ESR-Erythrocyte sedimentation rate, PJI-Periprosthetic joint infection

Number of patients with loosening	One stage revision	Two stage revision	P value
	n=25	n=23	-
PJI	2 of 25 (8%)	21 of 23 (91.3%)	P < 0.001
Sinus tract	0 of 25 (0%)	18 of 23 (78.2%)	P < 0.001
Elevated CRP>10 mg/L	8 of 25 (32%)	17 of 23 (73.9%)	P = 0.004
Elevated ESR>30 mm/H	7 of 25 (28%)	16 of 23 (69.5%)	P = 0.004
Preoperative positive synovial fluid culture	0 of 25(0%)	15 of 23 (65.2%)	P < 0.001
Elevated Fibrinogen>500 mg/dL	1 of 25 (4%)	6 of 23 (26%)	P = 0.030

Despite the fact that two cases of PJIs were misdiagnosed, we still conducted 23 septic revisions, the reason behind this number being that two cases of aseptic loosening were overdiagnosed as PJIs because of the presence of a turbid fluid when an articular capsule incision was performed. The liquid was sampled and sent to our microbiology department, where bacteriological results showed no signs of bacterial infection.

In only 15 patients out of 23 (65.2%) that underwent two-stage revision a pathogen bacteria was identified prior to surgery and no patient from the one-stage revision group had a positive preoperative bacterial culture, indicating a strong relationship between the positive culture and two-stage revision surgery (p<0.001). The relatively low identification percentage could be explained by the consumption of antibiotics before samples were obtained in some cases or the slow growth of biofilm-producing bacteria, exposing this technique to false negative results. Data is similar to the results of a published study by Daniel Karczewski et al. in 2018 concluding that a positive bacteriological sample prior to surgery is not essential in deciding the surgical option [15]. They managed to detect PJIs preoperatively and intraoperatively with great accuracy using the EBJIS (European Bone and Joint Infection Society) diagnostic protocol.

While CRP and ESR levels over the physiological limits were more frequent in the two-stage revision group (p=0.004), they were also elevated in the one-stage revision group, yielding a lower specificity when used to diagnose PJI. Elevated serum fibrinogen levels were encountered more in the two-stage revision group (p=0.030), but the frequency of this finding (six out of 23 patients) is too low to influence the diagnostic or treatment protocol.

To provide additional information about the specifics of periprosthetic joint infections, we illustrate some of the findings in Table 2.

**Table 2:** Comparative data of the population based on septic or aseptic loosening of the prosthesis BMI-Body Mass Index, CRP-C reactive protein, ESR-Erythrocyte sedimentation rate, WBC-White blood cells, PJI-Periprosthetic joint infection, AL-Aseptic loosening

Variables	PJI (n=23)	AL (n=25)
Male gender	10	15
Mean age (years)	72.47	67.53
BMI mean	26.98 kg/m^2^	28.19 kg/m^2^
Rural area	11	13
Smoking	13	5
Diabetes	10	5
Serum CRP mean	49.21 mg/L	7.68 mg/L
Serum ESR mean	55.34 mm/h	16.35 mm/h
Serum WBC mean	8.56 x 10^3^/uL	7.65 x 10^3^/uL
Serum fibrinogen mean	498.28 mg/dL	352.36 mg/dL
Time from primary arthroplasty mean(years)	5.11	11.20
Hospital stay mean(days)	29.91	13.93
Positive postoperative culture from tissue sample	13	2 (false positive)
Positive postoperative culture from sonication fluid	22	1 (false positive)
Positive histopathological exam for infection	22	0

We can notice an important difference between the two complications when we look at the time elapsed from primary arthroplasty until revision surgery. With a mean of 5.11 years (standard deviation 5.33; CI: 2.80 to 7.42) for PJI and 11.20 years (standard deviation 4.94; CI: 9.32 to 13.08) for aseptic loosening, a statistical difference was noted, PJI being correlated with a shorter period of time until revision surgery is performed (U=121.000, Z=-3.924, p<0.001).

PJI was also correlated with an increased hospital stay period when compared with the aseptic patients (U=92.000, Z=-4.454, p<0.001). The mean hospital stay period for septic patients was 29.91 days (standard deviation 15.98; CI: 23 to 36.83) and 13.93 days (standard deviation 7.30; CI: 11.15 to 16.71) for aseptic patients.

As seen in the table, postoperative tests like sonication fluid culture (sensitivity=95.6%, specificity=96%) or histopathological examination of the tissue samples (sensitivity 95.6%, specificity=100%) had the best accuracy in identifying PJI. They have an irreplaceable role in PJI diagnosing and management, detection of the causative bacteria being a key factor for successful treatment. However, we should keep in mind that these results become available only after surgery is performed, so they do not influence the preoperative treatment plan.

Highlighted cases

Cases Where Therapeutic Decision Was Made Intraoperative

In three cases that underwent two-stage revision surgery, the decision to deviate from the initial one-stage revision preoperative plan to this type of treatment and to implant a joint spacer in order to wait for the next surgical step was taken intraoperative, based on the macroscopic or microscopic examination of the periprosthetic tissue or fluid. As we stated previously, two cases where PJI was suspected were denied by the negative culture of the intraoperatively harvested samples or sonication fluid. However, the third one was confirmed as true PJI, although only slightly elevated CRP and ESR values (19 mg/L and 23 mm/H), negative culture from synovial fluid, pain, and radiological loosening were identified prior to surgery. These findings were insufficient to diagnose PJI.

Even though the paraclinical findings and the symptomatology of the patient prior to surgery were not enough to diagnose the PJI, the intraoperative decision to go with the two-stage revision technique was the correct choice in this case. We want to mention that the identification of coagulase-negative *Staphylococcus *was achieved only after the sonication of the explanted prosthesis, thus strengthening the idea of a chronic, low-virulence PJI. All three patients had a good outcome at six and twelve months after revision surgery, with no signs or symptoms of reinfection.

By deciding to undergo two-stage revision surgery for the two aseptic loosening cases that were misdiagnosed as PJIs, the total cost, hospital stay, and risks of additional anesthesia and surgery increased and the final treatment was delayed. However, the prognosis and evolution of those patients were favorable. All of the above should be avoided as much as possible by carefully analyzing patient history and clinical and paraclinical findings, but we should keep in mind that misdiagnosing a real PJI as aseptic loosening is a far more taxing mistake for the patient and healthcare system. We shall further elaborate on the two cases where PJI was underdiagnosed and aseptic revision surgery was performed.

Underdiagnosed PJI Cases

The first patient, a 71-year-old male with a BMI of 27.4 kg/m^2^, sought medical advice for pain in the left hip where radiological loosening of prosthesis was found. The primary arthroplasty of the hip was performed seven years ago and no history of infection of the affected joint prior to that was known. He did not have any other clinical signs of PJI, just pain, which can be common for aseptic loosening as well. Paraclinical tests indicated a serum CRP level of 1.9 mg/L, ESR level of 5 mm/H, leucocyte count of 7.4 *10³/uL, and a fibrinogen value of 309 mg/dl. These results once again sustained the assumption of aseptic loosening of the prosthesis. The surgical team proceeded with surgical revision of the prosthesis using the one-stage technique - removing the old prosthesis and implanting the new one in the same surgical time.

No signs of infection were detected during surgery, but additional periprosthetic tissue samples were collected for bacteriological and histopathological examination, and the explanted prosthesis was sent for sonication. The postoperative evolution of the patient was good. Bacteriological culture from the harvested tissue samples was negative, but histopathological examination identified a type 3 tissue (infectious and particle disease, mixed tissue) using Krenn-Morawietz periprosthetic tissue classification [16]. Unfortunately, the bacteriological culture results using the sonication fluid came back positive for *Citrobacter koseri *and *Staphylococcus aureus*. The patient initiated additional prolonged antibiotic therapy, but unfortunately, his evolution at six and twelve months was unfavorable after two more surgeries trying to eradicate the infection without success.

The second patient, a 74-year-old female with a BMI of 29.1 kg/m^2^, came to our hospital presenting pain and functional impairment of the left hip prosthesis. A shorter time period of four years had passed since the initial surgery. Inflammatory parameters indicated an elevated value of 50 mg/L for CRP and 51 mm/H for ESR, but with a negative preoperative synovial fluid culture and no history of infection, the attending doctor inclined towards aseptic one-stage revision. During surgery, no obvious signs of infection were detected and the surgical team proceeded with the initial plan of one-stage revision and harvested tissue samples. This time, both cultures inoculated with harvested samples and sonication fluid were positive for *Pseudomonas aeruginosa*.

The patient underwent double antibiotic therapy and was discharged with good general and local condition. Shortly after the discharge, the patient returned to our hospital with surgical wound dehiscence, and considering the recent implantation of the prosthesis, debridement and antibiotic therapy was attempted in order to retain the implant and treat the infection, but without success. The patient underwent two more surgeries to achieve sanitation of the periprosthetic joint infection.

## Discussion

When PJI suspicion is high but clear evidence is lacking, surgeons should be aware of the existence of another PJI version - culture-negative PJI. Diagnostic algorithms should not rely only on culture results or clinical findings when PJI is suspected. Culture-negative PJI was first described by Berbari et al. [17] as the lack of bacterial growth on aerobic or anaerobic cultures inoculated with periprosthetic tissue and the concomitant presence of periprosthetic purulence, inflammation at histopathological examination of tissue samples, or a sinus tract communicating with the prosthesis. The diagnosis of this type of PJI is not hindered by negative culture results. In most cases, these negative results occur due to the administration of antibiotics before sampling or the inability to detach biofilm-forming bacteria from the surface of the implant. However, a problem arises when suspicion of PJI is high, but there are no obvious clinical signs of infection, and cultures are negative. This was a situation we also faced, as described in the highlighted patients above.

In this particular case, it may be difficult to differentiate between an aseptic loosening (true negative culture) and a false negative result (PJI is present but cultures could not identify the pathogen bacteria) [18]. It is recommended that repeated sampling should be done and newer diagnostic techniques like 16s RNA analysis, sonication of the prosthesis, or other molecular techniques should be conducted in order to identify the microorganisms [19]. Current literature shows that the incidence of culture-negative PJI is between 7% to 15% [20]. In order to provide the best possible outcome when managing this type of PJI, a good interdisciplinary collaboration between microbiologists, radiologists, pathologists, and surgeons is key.

Acknowledging the impact of the presence of a sinus tract in PJI on the treatment algorithm and surgery outcome, a study conducted by Xu C et al. in 2019 managed to identify five risk factors that were related to this clinical sign [21]. The sinus tract may be more commonly encountered after hip surgery than the knee. The incidence of sinus tract is higher in patients who underwent multiple revision surgeries of the same joint, with some studies showing that this could be the result of compromised soft tissue and increased surgical dissection [22]. The third risk factor was hypo-albuminemia, with malnutrition having a negative influence on the wound-healing process, which could lead to reinfection. Studies have shown that malnutrition influences the synthesis of proteins like proteoglycans and collagen and the lack of essential nutrients for normal cellular function is related to poor immune system activity [23]. Fourth, smoking was another risk factor that could increase the chances of developing a sinus tract. Its effects on the cardiovascular system are well known. Chronic smoking is related to overall poor soft-tissue oxygenation, a condition amplified by nicotine-induced vasoconstriction in the capillary circulation [24]. Knowing that thyroid hormones regulate the immune system by influencing leukocyte migration, stimulating polymorphonuclear activity, and promoting antibody production and lymphocyte function, hypothyroidism has been identified as the fifth risk factor for PJI and sinus tract [25].

We can notice that in the tragic cases where periprosthetic joint infection was underdiagnosed, the evolution of the patients was unfavorable with the concomitant increase of hospital stay, number of surgeries needed to achieve sanitation of the infection, period of antibiotic therapy and morbidity, as well as the financial costs for the hospital managing those cases.

Although diagnostic criteria are in permanent development, today's standards are those proposed by societies like EBJIS, IDSA, and MSIS, which have great accuracy in detecting PJIs. The biggest efforts, however, are still focused on finding faster and cheaper diagnostic methods for low-grade chronic periprosthetic joint infections. These infections often do not present eloquent signs, symptoms, or paraclinical findings [26]. It is imperative to develop such novel methods, especially with preoperative or intraoperative applications, so that the surgical team can take the right actions and opt for the appropriate treatment prior to or during surgery. In both underdiagnosed cases presented above, the absence of obvious pathological findings prior to or during revision surgery led to substandard therapeutic decisions with important consequences.

The slow bacterial growth in standard bacteriological cultures could take up to 14 days [27], in some cases even exceeding the hospital stay period of the patient. While being the most used method to identify pathogen bacteria, when patients undergo antibiotic therapy or contamination occurs when transporting or manipulating the sample, it still has a high percentage of false positive/false negative results[28].

We should consider that the high percentage of patients who presented an active sinus tract at the initial examination in our hospital is mainly because they do not seek medical advice until the infection evolves to this stage, a demographic characteristic of the studied population. Defined as a draining tract communicating with the joint interior and the skin surface, the sinus tract is a major criterion for PJI diagnosis, keeping in mind the high specificity of the finding. However, more recent studies showed that its presence is also an independent risk factor for reinfection after revision [29].

Some other limitations of the current study are represented by the relatively low number of patients included and the lack of access to more expensive diagnostic methods like lateral flow test or ELISA (enzyme-linked immunosorbent assay) for detecting α-defensin, PET/CT (positron emission tomography) scan, PCR (polymerase chain reaction), NGS (Next Generation Sequencing), or inoculation of the samples on more specific cultures for periprosthetic joint infections [30].

## Conclusions

With the current study, we wanted to highlight that the clear signs of periprosthetic joint infection such as a sinus tract or purulence surrounding the prosthesis are still the main deciding factors for septic treatment in our clinic. 78.2% of the patients who underwent two-stage arthroplasty revision surgery had a sinus tract at the initial evaluation. While being a pathognomonic sign that manages to check all our diagnostic criteria requirements (fast preoperative diagnostic with maximum specificity), the sinus tract is unfortunately also a giveaway of the severity and unfavorable evolution of the infection. When PJI suspicion is high, medical staff should not completely exclude this diagnostic if microbiological cultures are negative or more evident clinical signs are absent and should keep in mind the possibility of a culture-negative PJI. In the cases where periprosthetic joint infections are misdiagnosed, the treatment is difficult and implies higher financial costs for the hospital and higher morbidity and longer hospital stays for the patient.
